# Comparison of BlockBuster® Laryngeal Mask Airway (LMA) and LMA® Supreme™ in Patients Undergoing General Anesthesia: A Prospective Study

**DOI:** 10.7759/cureus.112637

**Published:** 2026-07-14

**Authors:** Indu Gupta, Ankita Raj, Ankur Garg

**Affiliations:** 1 Department of Anesthesiology, Rohilkhand Medical College and Hospital, Bareilly, IND

**Keywords:** airway management, general anesthesia, laryngeal mask airway, oropharyngeal leak pressure, supraglottic airway devices

## Abstract

Introduction

Although second-generation supraglottic airway devices have been established in routine anesthetic practice for more than two decades, newer devices such as the BlockBuster® laryngeal mask airway (BlockBuster® LMA; Teleflex Medical, Westmeath, Ireland) have been developed with design modifications intended to improve airway sealing and facilitate tracheal intubation. However, direct comparative clinical evidence between the newer BlockBuster® LMA and the well-established LMA® Supreme™ (Tuoren Medical Device Co., Ltd., Henan, China) remains limited, particularly in adult patients undergoing routine general anesthesia. This study aimed to compare the efficacy and safety of the BlockBuster® LMA and LMA® Supreme™ in adults undergoing surgery under general anesthesia.

Materials and methods

This prospective study included 72 adult patients who underwent elective surgery under general anesthesia. The patients were allocated to either the BlockBuster® LMA group or the LMA® Supreme™ group, with 36 patients in each group. Device insertion characteristics, airway performance, hemodynamic parameters, gastric tube insertion characteristics, end-tidal carbon dioxide values, and postoperative airway-related complications were assessed. Statistical analysis was performed, and a p<0.05 was considered statistically significant.

Results

Baseline demographic and airway characteristics were comparable between the groups. The BlockBuster® LMA demonstrated significantly superior airway performance, with a higher oropharyngeal leak pressure (32.33 ± 1.97 cmH₂O vs. 25.58 ± 2.41 cmH₂O; p<0.001). Easy insertion was achieved in 28 (38.9%) and 24 (33.3%) patients in the BlockBuster® LMA and LMA® Supreme™ groups, respectively. Airway manipulation was required in 14 (38.9%) and 20 (55.6%) patients, respectively. Easy gastric tube insertion was observed in 33 (45.8%) and 31 (43.0%) patients in the BlockBuster® LMA and LMA® Supreme™ groups, respectively. The number of insertion attempts, gastric tube insertion time, end-tidal carbon dioxide values, and hemodynamic parameters were comparable between the groups. Postoperative airway morbidity and device-related complications were infrequent and showed no significant intergroup differences in the present study.

Conclusion

Both devices provided effective and safe airway management during general anesthesia. However, the BlockBuster® LMA demonstrated higher oropharyngeal leak pressure, indicating improved airway sealing performance, while maintaining a safety profile comparable to that of the LMA® Supreme™. These findings support the use of both LMAs as reliable second-generation supraglottic airway devices in routine anesthesia practice.

## Introduction

Airway management remains one of the most fundamental responsibilities of the anesthesiologist and plays a crucial role in ensuring patient safety during general anesthesia [1]. The maintenance of a patent airway is essential for adequate oxygenation, ventilation, and delivery of anesthetic agents throughout surgical procedures. While endotracheal intubation has traditionally been considered the gold standard for airway control, it is associated with several limitations, including the requirement for greater technical expertise, increased sympathetic stimulation, and postoperative airway-related complications such as sore throat, coughing, and mucosal trauma [2]. Consequently, supraglottic airway (SGA) devices have emerged as valuable alternatives for airway management in appropriately selected patients [3].

Second-generation SGA devices have been specifically designed to improve airway sealing, facilitate positive pressure ventilation, and enhance protection against aspiration by incorporating gastric drainage channels. Among these devices, the laryngeal mask airway (LMA) Supreme (LMA® Supreme™) and BlockBuster® LMA have gained popularity because of their ease of insertion, reliable ventilation characteristics, and favorable safety profiles [4,5]. The LMA® Supreme™ is a single-use, anatomically curved device that provides effective airway sealing and facilitates gastric access. Previous studies have reported high first-attempt insertion success rates, satisfactory oropharyngeal leak pressures, and low rates of airway complications with its use [4,6].

The BlockBuster® LMA is a newer second-generation SGA device designed to provide improved airway sealing and facilitate endotracheal intubation when required. Its anatomically contoured airway tube, soft silicone cuff, and enhanced sealing characteristics have been shown to provide effective ventilation and favorable clinical performance [5]. Several studies have suggested that the BlockBuster® LMA may offer higher oropharyngeal leak pressures and improved insertion characteristics than other SGAs [5,7].

Although both devices are widely used in contemporary anesthetic practice, the available evidence comparing their clinical performance remains limited, particularly in routine surgical patients undergoing general anesthesia. A comparative evaluation of insertion characteristics, airway sealing efficacy, hemodynamic responses, gastric tube placement, and postoperative airway morbidity is important for identifying the most suitable device for clinical practice. Therefore, the present prospective study was conducted to evaluate and compare the efficacy and safety of the BlockBuster® LMA and LMA® Supreme™ in adult patients undergoing surgical procedures under general anesthesia. The objectives were to compare the device insertion characteristics, airway performance, hemodynamic parameters, ease of gastric tube insertion, and postoperative pharyngolaryngeal complications associated with the use of the BlockBuster® LMA and LMA® Supreme™.

## Materials and methods

This prospective study was conducted at the Department of Anesthesiology, Rohilkhand Medical College and Hospital, Bareilly, Uttar Pradesh, India, following approval from the Institutional Ethics Committee (IEC/RMCH/01/2024/APR dated June 11, 2024). Adult patients scheduled to undergo elective surgical procedures under general anesthesia between June 2024 and May 2025 were screened for eligibility. Written informed consent was obtained from all participants prior to enrolment. The study was conducted in accordance with the ethical principles of the Declaration of Helsinki and the Good Clinical Practice guidelines [8].

Patients received either the BlockBuster® LMA or the LMA® Supreme™ according to the treating consultant anesthesiologist's routine clinical judgment, device availability, and departmental practice. No random sequence generation, allocation concealment, or intervention assignment by the investigators was performed. The investigators prospectively recorded and compared the perioperative outcomes associated with the two routinely used SGA devices.

The sample size was determined using G*Power software version 3.1.9.7 (Heinrich Heine University, Düsseldorf, Germany). Based on previously reported differences in oropharyngeal leak pressure between second-generation SGA devices, an effect size of 0.67, a two-sided alpha level of 0.05, and a statistical power of 80% were considered [9]. The minimum required sample size was calculated as 72 participants; therefore, 72 patients were included, with 36 patients evaluated for each airway device.

Patients aged 18-65 years, belonging to the American Society of Anesthesiologists (ASA) physical status I or II [10], having a body mass index below 26 kg/m², Mallampati grade I or II airway assessment [11], and adequate head and neck mobility were considered eligible for inclusion in the study. Patients with anticipated difficult airway, difficult mask ventilation, cervical spine instability, temporomandibular joint disorders, gastroesophageal reflux disease, increased risk of aspiration, pregnancy, previous pharyngeal or laryngeal surgery, thoracotomy, requirement for lateral or prone positioning, or expected surgical duration exceeding four hours were excluded.

All patients underwent a detailed pre-anesthetic evaluation, including clinical examination and routine laboratory investigations. Standard fasting guidelines recommended by the ASA were followed. Upon arrival in the operating room, routine monitoring consisting of electrocardiography, non-invasive blood pressure (BP) monitoring, pulse oximetry, and capnography was established using a multiparameter monitor. Baseline heart rate, systolic BP, diastolic BP, mean arterial pressure, and peripheral oxygen saturation were recorded before the induction of anesthesia.

Premedication consisted of intravenous glycopyrrolate (0.004 mg/kg), midazolam (0.02 mg/kg), and butorphanol (0.02 mg/kg). Following preoxygenation with 100% oxygen for 3 min, anesthesia was induced using intravenous propofol (2 mg/kg). After confirmation of adequate mask ventilation, neuromuscular blockade was achieved with intravenous vecuronium bromide (0.1 mg/kg), and controlled ventilation was initiated. Following adequate jaw relaxation, airway management was performed using either the BlockBuster® LMA (Tuoren Medical Device Co., Ltd., Henan, China) or LMA® Supreme™ (Teleflex Medical, Westmeath, Ireland). Device size selection was based on the manufacturer's recommendations according to the patient’s body weight. Before insertion, each device was inspected for integrity and lubricated with a sterile water-soluble lubricant applied to the cuff’s posterior surface.

Insertion was performed by an experienced anesthesiologist using the standard midline insertion technique with the patient in the supine position and the head maintained in a neutral position. For the LMA® Supreme™, the device was advanced along the hard palate until resistance was encountered in the hypopharynx [4]. The BlockBuster® LMA was inserted using a similar midline approach and advanced until optimal seating in the hypopharynx was achieved [5]. Following insertion, the cuffs of both devices were inflated to a cuff pressure of 30 cmH₂O using a cuff pressure manometer (Portex, Smiths Medical, Minneapolis, Minnesota, USA).

The airway device was connected to the anesthesia breathing circuit, and correct placement was confirmed by bilateral chest expansion, equal breath sounds on auscultation, peripheral oxygen saturation greater than 95%, appearance of a square-wave capnographic trace, and adequate tidal volume delivery. In cases where adequate sealing or ventilation was not achieved after initial insertion, standard corrective manoeuvres, including jaw thrust and minor repositioning of the laryngeal mask airway, were performed before proceeding with outcome measurements. Oropharyngeal leak pressure was measured only after satisfactory device positioning had been confirmed by bilateral chest expansion, an adequate capnographic waveform, and effective ventilation. If satisfactory placement could not be achieved after a maximum of three insertion attempts, the device was considered a failed insertion and an alternative airway management strategy would have been instituted according to standard clinical practice. However, no such cases occurred during the study.

The primary outcome measure was the oropharyngeal leak pressure. The oropharyngeal leak pressure was measured by closing the adjustable pressure-limiting valve to a maximum pressure of 40 cmH₂O with a fresh gas flow of 3 L/min and recording the airway pressure at equilibrium or at the point of an audible leak. Secondary outcome measures included insertion time, number of insertion attempts, ease of insertion, airway manipulation requirement, ease and duration of gastric tube insertion, end-tidal carbon dioxide values, hemodynamic parameters, and perioperative airway-related complications. Device insertion time was defined as the interval between picking up the device and obtaining a satisfactory square-wave capnographic trace [12]. Ease of insertion was classified as easy when the device was successfully inserted without the need for additional airway maneuvres and as fair when minor corrective maneuvres, such as jaw thrust or positional adjustment, were required to achieve effective ventilation.

Subsequently, a lubricated 10-French gastric tube was inserted through the gastric drainage channel of the respective device, and successful placement was confirmed by auscultation of the epigastric region. Anesthesia was maintained using oxygen, nitrous oxide, and isoflurane, with supplemental vecuronium administered as required. Hemodynamic variables were recorded at baseline, immediately after device insertion, and every 15 minutes thereafter until completion of surgery. For statistical analysis, the values recorded at 15 minutes after device insertion were used as the intraoperative measurements. 

At the end of the surgery, residual neuromuscular blockade was reversed, and the SGA device was removed after the return of adequate spontaneous respiration and protective airway reflexes. The device was inspected for blood staining, and the patients were evaluated for postoperative pharyngolaryngeal morbidity, including sore throat, hoarseness, cough, laryngospasm, bronchospasm, and clinical evidence of aspiration. Postoperative sore throat was assessed using a four-point ordinal scale immediately after device removal and at 30 min, two hours, and four hours postoperatively. Postoperative hemodynamic parameters were recorded 30 minutes after removal of the SGA device.

Statistical analyses were performed using IBM SPSS Statistics for Windows, Version 25 (Released 2017; IBM Corp., Armonk, New York, United States). The normality of the continuous variables was assessed using the Shapiro-Wilk test. Continuous variables are expressed as mean ± standard deviation, whereas categorical variables are presented as frequency and percentage. Comparisons between groups were performed using the independent-samples t-test for normally distributed continuous variables and the chi-square test or Fisher’s exact test for categorical variables, as appropriate. All statistical tests were two-tailed, and a p<0.05 was considered statistically significant. Effect sizes and 95% confidence intervals (CIs) were calculated, where applicable.

## Results

A total of 72 patients were evaluated, comprising 36 (50%) and 36 (50%) patients in the BlockBuster® LMA and LMA® Supreme™ groups, respectively. The baseline demographic, clinical, and laboratory characteristics are presented in Tables 1, 2.

**Table 1:** Baseline demographic, clinical, and laboratory characteristics of the study participants Group 1: BlockBuster® LMA; Group 2: LMA® Supreme™. Data are presented as mean ± standard deviation (SD) with 95% confidence interval (CI), Comparisons between groups were performed using the independent samples t-test, Effect size is reported as Cohen's d, CI: confidence interval, *p<0.05 was considered statistically significant.

Parameter	Group 1 (n=36), Mean ± SD	CI at 95%	Group 2 (n=36), Mean ± SD	CI at 95%	t statistic (df=70)	p-value	Cohen's d
Age (years)	34.03 ± 13.71	29.39-38.67	36.86 ± 12.33	32.69-41.03	-0.92	0.360	-0.22
Weight (kg)	54.39 ± 10.12	50.97-57.81	53.53 ± 8.17	50.76-56.29	0.39	0.692	0.09
Height (cm)	160.97 ± 5.20	159.21-162.73	161.92 ± 4.25	160.48-163.35	-0.84	0.401	-0.19
Hemoglobin (g/dL)	12.11 ± 1.09	11.74-12.48	12.24 ± 1.04	11.88-12.59	-0.50	0.613	-0.12
Total leukocyte count (/mm³)	9358.44 ± 2075.71	8656.12-10060.77	7700.31 ± 2712.82	6782.42-8618.19	2.91	0.005*	0.68
Neutrophils (%)	62.11 ± 7.77	59.48-64.74	60.83 ± 7.95	58.14-63.52	0.69	0.493	0.16
Lymphocytes (%)	30.25 ± 8.99	27.21-33.29	32.47 ± 9.66	29.20-35.74	-1.01	0.316	-0.23
Eosinophils (%)	3.31 ± 2.11	2.59-4.02	3.58 ± 1.87	2.95-4.22	-0.59	0.556	-0.13
Monocytes (%)	3.67 ± 2.00	2.99-4.34	2.56 ± 1.72	1.98-3.14	2.53	0.014*	0.59
Basophils (%)	0.58 ± 0.97	0.26-0.91	0.72 ± 1.03	0.37-1.07	-0.58	0.558	-0.13
Blood sugar (mg/dL)	112.31 ± 38.82	99.17-125.44	119.94 ± 18.42	113.71-126.18	-1.06	0.290	-0.25
Blood urea (mg/dL)	20.98 ± 6.64	18.73-23.23	25.19 ± 5.19	23.44-26.95	-3.01	0.004*	-0.70
Serum creatinine (mg/dL)	0.83 ± 0.22	0.76-0.91	0.93 ± 0.24	0.85-1.01	-1.83	0.071	-0.43
Sodium (mEq/L)	137.94 ± 2.78	137.01-138.88	139.50 ± 3.86	138.20-140.81	-1.96	0.054	-0.46
Potassium (mEq/L)	4.14 ± 0.41	4.00-4.28	4.33 ± 0.49	4.17-4.50	-1.81	0.075	-0.42
Chloride (mEq/L)	101.14 ± 2.52	100.29-101.99	99.53 ± 1.91	98.88-100.17	3.06	0.003*	0.72
Prothrombin time (s)	12.86 ± 1.37	12.39-13.32	12.69 ± 1.26	12.27-13.12	0.51	0.605	0.12

**Table 2:** Comparison of baseline categorical demographic and clinical characteristics between the study groups. Group 1: BlockBuster® LMA; Group 2: LMA® Supreme™. Data are presented as frequency (n) and percentage (%), Comparisons between groups were performed using the chi-square test, Effect size is reported as Cramer's V, p<0.05 was considered statistically significant.

Parameter		Group 1 (n=36), n (%)	Group 2 (n=36), n (%)	χ² statistic (df)	p-value	Cramer's V
Age group	1 to 20	9 (12.5)	3 (4.2)	3.62 (2)	0.163	0.22
	21 to 40	15 (20.8)	19 (26.4)			
	41 to 60	12 (16.7)	14 (19.4)			
Mallampati grading	1	14 (19.4)	14 (19.4)	1.02 (2)	0.601	0.12
	2	22 (30.6)	21 (29.2)			
	3	0 (0)	1 (1.4)			

The majority of participants belonged to the 21-40-year age category. Mallampati grade II was the most common airway classification observed in both groups. Overall, the groups demonstrated comparable baseline characteristics.

As shown in Table 3, the BlockBuster® LMA demonstrated superior airway performance compared to the LMA® Supreme™.

**Table 3:** Comparison of airway device insertion characteristics and airway performance between the study groups Group 1: BlockBuster® LMA; Group 2: LMA® Supreme™. Data are presented as mean ± standard deviation (SD) with 95% confidence interval (CI), Comparisons between groups were performed using the independent samples t-test, Effect size is reported as Cohen's d, *p<0.05 was considered statistically significant.

Parameter	Group 1 (n=36), Mean ± SD	CI at 95%	Group 2 (n=36), Mean ± SD	CI at 95%	t statistic (df=70)	p-value	Cohen's d
Time taken for insertion (s)	13.19 ± 2.51	12.34-14.04	10.97 ± 2.08	10.27-11.67	-4.08	<0.001*	-0.96
Number of attempts	1.17 ± 0.38	1.04-1.30	1.11 ± 0.32	1.00-1.22	0.67	0.502	0.15
Oropharyngeal leak pressure (cmH₂O)	32.33 ± 1.97	31.67-33.00	25.58 ± 2.41	24.77-26.40	13.01	<0.001*	3.06
Time for gastric tube insertion (s)	10.06 ± 1.69	9.48-10.63	10.03 ± 1.52	9.51-10.54	0.07	0.942	0.02
End-tidal carbon dioxide (ETCO₂, mmHg)	40.81 ± 2.27	40.04-41.57	40.64 ± 2.97	39.64-41.64	0.26	0.790	0.06

The mean insertion time was significantly lower in the LMA® Supreme™ group (10.97 ± 2.08 s) than in the BlockBuster® LMA group (13.19 ± 2.51 s; p<0.001). Furthermore, the BlockBuster® LMA achieved a significantly higher oropharyngeal leak pressure (32.33 ± 1.97 cmH₂O vs. 25.58 ± 2.41 cmH₂O, p<0.001). No significant differences were observed in the number of insertion attempts, gastric tube insertion time, or end-tidal carbon dioxide values (p>0.05 for all).

A comparison of the categorical airway performance parameters is presented in Table 4.

**Table 4:** Comparison of airway device insertion success and procedural characteristics between the study groups Group 1: BlockBuster® LMA; Group 2: LMA® Supreme™. Data are presented as frequency (n) and percentage (%), Comparisons between groups were performed using the chi-square test, Effect size is reported as Cramer's V, p<0.05 was considered statistically significant.

Parameter		Group 1 (n=36), n (%)	Group 2 (n=36), n (%)	χ² statistic (df)	p-value	Cramer's V
Ease of insertion	Easy	28 (38.9)	24 (33.3)	1.11 (1)	0.293	0.12
	Fair	8 (11.1)	12 (16.7)			
Airway manipulation required	No	22 (30.6)	16 (22.2)	2.01 (1)	0.157	0.17
	Yes	14 (19.4)	20 (27.8)			
Ease of gastric tube insertion	Easy	33 (45.8)	31 (43.1)	0.56 (1)	0.453	0.09
	Fair	3 (4.2)	5 (6.9)			

Easy insertion was achieved in 28 (38.9%) and 24 (33.3 %) patients in the BlockBuster® LMA and LMA® Supreme™ groups, respectively. Airway manipulation was required in 14 (19.4%) and 20 (27.8%) patients. Easy gastric tube insertion was observed in 33 (45.8%) and 31 (43.1%) patients in the BlockBuster® LMA and LMA® Supreme™ groups, respectively. However, none of these differences were statistically significant (p > 0.05).

The hemodynamic parameters are summarized in Table 5.

**Table 5:** Comparison of hemodynamic and oxygenation parameters at different perioperative time points between the study groups Group 1: BlockBuster® LMA; Group 2: LMA® Supreme™. Data are presented as mean ± standard deviation (SD) with 95% confidence interval (CI), Comparisons between groups were performed using the independent samples t-test, Effect size is reported as Cohen's d, BP: blood pressure, *p<0.05 was considered statistically significant.

Parameter	Category	Group 1 (n=36), mean± SD	CI at 95%	Group 2 (n=36), mean± SD	CI at 95%	t statistic (df=70)	p-value	Cohen’s d
Heart rate (per minute)	Baseline	87.75 ± 9.89	84.40-91.10	79.81 ± 10.84	76.14-83.48	3.24	0.002*	0.76
	After induction	88.25 ± 6.11	86.18-90.32	83.42 ± 5.74	81.47-85.36	3.45	<0.001*	0.81
	Immediately after device insertion	87.47 ± 4.60	85.92-89.03	100.17 ± 6.23	98.06-102.28	-9.83	<0.001*	-2.31
	15 minutes after device insertion (Intraoperative)	86.33 ± 5.90	84.34-88.33	88.25 ± 5.72	86.32-90.19	-1.40	0.166	-0.33
	Immediately before device removal	81.61 ± 5.64	79.70-83.52	85.97 ± 4.88	84.32-87.62	-3.50	<0.001*	-0.82
	Immediately after device removal (Postoperative)	81.00 ± 4.79	79.38-82.62	82.89 ± 5.73	80.95-84.83	-1.51	0.133	-0.35
BP (systolic)	Baseline	119.22 ± 10.32	115.73-122.72	119.75 ± 9.59	116.51-123.00	-0.22	0.823	-0.05
	After induction	122.44 ± 6.64	120.20-124.69	120.36 ± 7.12	117.95-122.77	1.28	0.203	0.30
	Immediately after device insertion	120.28 ± 8.59	117.37-123.18	120.14 ± 8.19	117.37-122.91	0.07	0.944	0.01
	15 minutes after device insertion (Intraoperative)	122.75 ± 7.22	120.31-125.19	121.19 ± 7.79	118.56-123.83	0.87	0.382	0.20
	Immediately before device removal	118.47 ± 7.08	116.08-120.87	131.00 ± 8.80	128.02-133.98	-6.65	<0.001*	-1.56
	Immediately after device removal (Postoperative)	120.14 ± 8.19	117.37-122.91	118.00 ± 7.20	115.56-120.44	1.17	0.243	0.27
BP (diastolic) baseline	Baseline	76.94 ± 7.91	74.27-79.62	75.28 ± 7.25	72.83-77.73	0.93	0.355	0.22
	After induction	76.42 ± 5.08	74.70-78.14	76.42 ± 5.08	74.70-78.14	0.00	1.000	0.00
	Immediately after device insertion	76.92 ± 4.88	75.26-78.57	75.58 ± 4.81	73.96-77.21	1.16	0.247	0.27
	15 minutes after device insertion (Intraoperative)	75.97 ± 5.39	74.15-77.79	75.19 ± 4.48	73.68-76.71	0.66	0.508	0.15
	Immediately before device removal	74.86 ± 5.30	73.07-76.65	75.14 ± 5.18	73.39-76.89	-0.22	0.823	-0.05
	Immediately after device removal (Postoperative)	76.28 ± 5.48	74.42-78.13	82.06 ± 5.17	80.31-83.80	-4.60	<0.001*	-1.08
MAP baseline	Baseline	91.04 ± 7.43	88.52-93.55	90.10 ± 6.90	87.77-92.44	0.55	0.582	0.13
	After induction	91.76 ± 3.66	90.52-93.00	91.07 ± 4.16	89.66-92.47	0.75	0.455	0.17
	Immediately after device insertion	91.37 ± 4.19	89.95-92.79	90.44 ± 4.13	89.04-91.83	0.95	0.344	0.22
	15 minutes after device insertion (Intraoperative)	91.57 ± 3.94	90.23-92.90	90.53 ± 3.73	89.27-91.79	1.14	0.256	0.27
	Immediately before device removal	89.40 ± 4.36	87.92-90.87	93.76 ± 4.66	92.18-95.34	-4.10	<0.001*	-0.96
	Immediately after device removal (Postoperative)	90.90 ± 4.27	89.45-92.34	94.04 ± 4.05	92.67-95.41	-3.20	0.002*	-0.75
SpO2 baseline	Baseline	98.44 ± 0.94	98.13-98.76	98.58 ± 1.03	98.24-98.93	-0.59	0.551	-0.14
	After induction	98.42 ± 0.91	98.13-98.76	98.58 ± 1.02	98.24-98.93	-0.48	0.628	-0.11
	15 minutes after device insertion (Intraoperative)	98.42 ± 0.91	98.11-98.72	98.53 ± 1.03	98.18-98.88	0.89	0.373	0.21
	Immediately before device removal	98.31 ± 1.06	98.11-98.72	98.53 ± 1.03	98.18-98.88	-1.85	0.068	-0.43
	Immediately after device removal (Postoperative)	98.42 ± 1.05	97.95-98.67	98.75 ± 0.97	98.42-99.08	-0.88	0.378	-0.20

Both devices maintained satisfactory intraoperative and cardiovascular stability. Although statistically significant differences in heart rate and systolic blood pressure were observed at selected time points, oxygenation and ventilation remained within clinically acceptable ranges in both groups throughout the procedure.

The postoperative outcomes and device-related complications are shown in Table 6.

**Table 6:** Comparison of airway-related complications and postoperative morbidity between the study groups Group 1: BlockBuster® LMA; Group 2: LMA® Supreme™. Data are presented as frequency (n) and percentage (%), Comparisons between groups were performed using the chi-square test, Effect size is reported as Cramer's V, p<0.05 was considered statistically significant.

Parameters	Categories	Group 1, n (%)	Group 2, n (%)	χ² statistic (df)	p-value	Cramer's V
Blood stain on airway device	Absent	25 (34.7)	19 (26.4)	2.10 (1)	0.147	0.17
	Present	11 (15.3)	17 (23.6)			
Sore throat	Immediate	16 (22.2)	26 (36.1)	3.13 (3)	0.312	0.16
	30 min	12 (16.7)	18 (25.0)			
	2 hours	3 (4.2)	4 (5.5)			
	Postoperative	1 (1.4)	0 (0)			
Postoperative pharyngolaryngeal morbidity	No	30 (41.7)	27 (37.5)	0.76 (1)	0.384	0.10
	Yes	6 (8.3)	9 (12.5)			

The incidence of postoperative pharyngolaryngeal morbidity was low in both groups. Postoperative pharyngolaryngeal morbidity was defined as the occurrence of sore throat, hoarseness, cough, or laryngospasm following removal of the SGA device. Blood staining of the device and postoperative sore throat occurred infrequently, with no significant intergroup differences. No major airway-related adverse events, including aspiration, bronchospasm, or laryngospasm, were observed. Overall, both devices demonstrated a favorable safety profile; however, the BlockBuster® LMA provided superior insertion characteristics and significantly higher airway-sealing pressure.

## Discussion

The present prospective comparative study evaluated the clinical performance and safety of the BlockBuster® LMA and the LMA® Supreme™ in adult patients undergoing elective surgical procedures under general anesthesia. The findings demonstrated that both SGA devices provided effective airway maintenance with high insertion success rates and satisfactory perioperative safety profiles. However, the BlockBuster® LMA exhibited superior airway performance, particularly with respect to insertion characteristics and oropharyngeal leak pressures. Baseline demographic and airway characteristics were comparable between the two groups, indicating that the observed differences in airway outcomes were primarily attributable to device-related factors rather than patient-related confounders. The similarity in age distribution, anthropometric parameters, and Mallampati grading ensured an equitable comparison between the study groups.

One of the notable findings of the present study was the shorter insertion time observed with the LMA® Supreme™ compared with the BlockBuster® LMA. This finding may be attributed to the preformed anatomically curved airway tube and relatively rigid structure of the LMA® Supreme™ device, which facilitates rapid advancement along the palatopharyngeal curve and allows quicker attainment of an effective airway seal. A shorter insertion time is clinically advantageous as it reduces the duration of airway manipulation and minimizes apnea time during airway establishment. The high first-attempt insertion success and ease of placement associated with the LMA® Supreme™ device may have further contributed to its superior insertion performance. Similar findings have been reported by Verghese and Verghese et al. [13], Hosten et al. [14], and Singh et al. [15], who demonstrated that the LMA® Supreme™ could be inserted rapidly and reliably with high first-pass success rates, making it a practical and user-friendly SGA device for routine anesthesia practice.

Another important observation was the significantly higher oropharyngeal leak pressure achieved with the BlockBuster® LMA device. Oropharyngeal leak pressure is widely regarded as an indicator of airway sealing efficacy and reflects the ability of a SGA device to provide effective positive pressure ventilation [16]. The superior leak pressure observed in the present study may be attributed to the unique cuff configuration and anatomical design of the BlockBuster® LMA, which allows for improved adaptation to the perilaryngeal structures [5]. Higher sealing pressures are particularly beneficial during controlled ventilation because they reduce the likelihood of gas leakage and gastric insufflation. The present findings are consistent with those reported by Gao et al. [17], and Endigeri et al. [18], all of whom demonstrated significantly higher oropharyngeal leak pressures with the BlockBuster® LMA device.

Despite these differences, the number of insertion attempts and ease of insertion were comparable between the two groups. Most devices were successfully inserted on the first attempt, indicating that both airway devices are user-friendly and can be effectively deployed by experienced anesthesiologists. Similar insertion success rates have been previously reported by Verghese and Verghese et al. [13] and Hosten et al. [14], who observed high first-pass insertion rates with the second-generation SGA devices. The requirement for additional airway manipulation was low in both groups and did not differ significantly between the groups. This finding suggests that both devices provide satisfactory anatomical alignment of the laryngeal inlet. Likewise, gastric tube placement was successful in the majority of patients, irrespective of the device used. The integrated gastric drainage channels present in both airway devices likely contributed to the ease of gastric tube insertion and enhanced protection against gastric insufflation and aspiration [19].

The hemodynamic profile remained stable throughout the perioperative period in both groups. Although minor statistical differences were observed at the selected time points, these variations were not clinically significant. SGA devices generally provoke less sympathetic stimulation than endotracheal intubation because they avoid direct laryngoscopy and tracheal instrumentation [20]. Both the BlockBuster® LMA and LMA® Supreme™ were associated with satisfactory cardiovascular stability during insertion and removal. Similar observations have been reported by Karaaslan et al. [21] and Park et al. [22]. In the present study, postoperative airway morbidity was minimal. The incidence of sore throat, blood staining of the device, and other airway-related complications were low and comparable between the groups. The soft cuff material and atraumatic design of both devices likely contributed to the favorable postoperative outcomes. Previous investigations by Endigeri et al. [18], and Gao et al. [17] also reported low complication rates associated with the BlockBuster® LMA and LMA® Supreme™.

Clinical implications

The findings of this study support the use of both BlockBuster® LMA and LMA® Supreme™ as reliable second-generation SGA devices in adult patients undergoing elective surgery under general anesthesia. However, the superior oropharyngeal leak pressure observed with the BlockBuster® LMA device may make it a preferable option in situations where effective positive-pressure ventilation are required. Improved airway sealing may enhance ventilation efficiency and reduce the risk of perioperative gas leakage.

Limitations

Certain limitations should be considered when interpreting the results of the present study. First, the study was conducted at a single tertiary care center with a relatively modest sample size, which may limit the generalizability of the findings. Second, only adult patients with anticipated normal airways and ASA physical status I-II were included; therefore, the results may not be directly applicable to patients with difficult airways, obesity, or significant systemic comorbidities. Third, all airway insertions were performed by experienced anesthesiologists to minimize operator-related variability. Consequently, the findings reflect device performance under standardized conditions and may not fully capture performance across operators with varying levels of experience. Finally, fibreoptic assessment of device positioning was not performed, which could have provided additional information regarding anatomical placement and airway alignment of the device. In addition, because treatment allocation was based on routine clinical practice rather than randomization, the study is susceptible to selection bias and residual confounding despite comparable baseline characteristics between the groups.

## Conclusions

Both the BlockBuster® LMA and LMA® Supreme™ are safe and effective SGA devices for adult patients undergoing elective surgical procedures under general anesthesia. However, the BlockBuster® LMA demonstrated superior clinical performance, characterized by a significantly higher oropharyngeal leak pressure, indicating improved airway sealing. Despite these advantages, both devices provided comparable insertion success rates, ease of insertion, hemodynamic stability, ventilation adequacy, and low postoperative airway morbidity, supporting their suitability for routine anesthesia practice.
